# Neuroprotective effects of quercetin on cerebral vasospasm following experimental subarachnoid haemorrhage in rats

**DOI:** 10.3906/sag-1904-207

**Published:** 2020-06-23

**Authors:** Şanser GÜL, Evren AYDOĞMUŞ, Burak BAHADIR, Çağatay BÜYÜKUYSAL, Berrak GÜVEN

**Affiliations:** 1 Department of Neurosurgery, Faculty of Medicine, Bülent Ecevit University, Zonguldak Turkey; 2 Department of Neurosurgery, Kartal Dr. Lütfi Kırdar Training and Research Hospital, İstanbul Turkey; 3 Department of Pathology, Faculty of Medicine, Bülent Ecevit University, Zonguldak Turkey; 4 Department of Biostatistics, Faculty of Medicine, Bülent Ecevit University, Zonguldak Turkey; 5 Department of Biochemistry, Faculty of Medicine, Bülent Ecevit University, Zonguldak Turkey

**Keywords:** Quercetin, cerebral vasospasm, subarachnoid haemorrhage

## Abstract

**Background/aim:**

We examined the protective effects of the natural flavonoid, quercetin, against cerebral vasospasm in an experimental rat subarachnoid haemorrhage (SAH) model.

**Materials and methods:**

Thirty-eight albino Wistar rats were divided into five groups as follows: group 1 (G1, *n*=8), no experimental intervention; group 2 (G2, *n*=8), subarachnoid physiological saline; group 3 (G3, *n*=8), SAH; group 4 (G4, *n*=7) SAH and low-dose (10 mg/kg) quercetin treatment; group 5 (G5, *n*=7), SAH and high-dose (50 mg/kg) quercetin treatment. Subarachnoid haemorrhage was induced by injection of 0.15 cc of autologous blood taken from the tail artery into the cisterna magna from the craniocervical junction and basilar arteries and blood samples were taken for biochemical and histopathological analyses.

**Results:**

Malondialdehyde (MDA) levels were significantly higher in G2 and G3 than in G1 (P < 0.05). Significant decreases in MDA were observed in G4 and G5 compared with G2 (P < 0.05, G4–G2; P < 0.05, G5–G2). There were no significant differences between G2 and G3 or among G1, G4, and G5. No statistically significant differences were found in total antioxidant capacity between the groups (P > 0.05). There were no significant differences in basilar artery (BA) wall thickness between G3 and G4 or between G3 and G5, but G4 and G5 showed greater luminal diameters than G3 (P < 0.05). There were no significant differences in BA thickness or luminal diameter between G4 and G5.

**Conclusion:**

Our results suggested that quercetin may be beneficial in SAH therapy by preventing vasospasm.

## 1. Introduction

Cerebral vasospasm (CV) is the most important cause of morbidity and mortality following subarachnoid haemorrhage (SAH) [1]. The aim of SAH treatment is to prevent secondary brain injury by optimising cerebral blood blow, reducing cerebral metabolic demand, and avoiding hypoxia [2]. Many pharmacological agents have been utilised for treatment of CV in both clinical and experimental studies, including free radical scavengers, vasodilators such as nitric oxide (NO), endothelin antagonists, glutamate antagonists and protein kinase inhibitors [3]. The natural flavonoid, quercetin (3,3′,4′,5,-pentahydroxyl-flavone), is present in high concentrations in fruits and vegetables, such as tea, apples, onions, potatoes, broccoli, and peanuts [4]. As a strong antioxidant and radical scavenger, quercetin has been shown to have beneficial effects in treatment of brain oedema and neuronal damage following SAH in experimental models by protecting against oxidative stress, and it may achieve efficient therapeutic levels in the brain as it can pass the blood–brain barrier in contrast to many other antioxidant agents [3,4]. 

The present study was designed to investigate the potential neuroprotective effects of quercetin on CV using an experimental rat model of subarachnoid haemorrhage, and to evaluate the cellular changes through biochemical, pathological, and histomorphometric analyses. 

## 2. Materials and methods

The experimental protocol was approved by the Ethics Committee of Zonguldak Bülent Ecevit University. The study was conducted on thirty-eight male albino Wistar rats, 4 months old, each weighing between 300 and 350 g, at the Experimental Surgery, Research and Animal Laboratory of the Faculty of Medicine, Zonguldak Bülent Ecevit University. All rats were kept under appropriate environmental conditions and they were divided into five groups as follows: group 1 (G1, *n*=8), no experimental intervention; group 2 (G2, *n*=8), subarachnoid physiological saline; group 3 (G3, *n*=8), SAH; group 4 (G4, *n*=7) SAH and low-dose (10 mg/kg) quercetin treatment; group 5 (G5, *n*=7), SAH and high-dose (50 mg/kg) quercetin treatment.

The experimental model was constructed as described previously [1,3,5]. Rats were anaesthetised by intraperitoneal injection of ketamine (60 mg/kg) and xylazine (10 mg/kg). A small suboccipital incision was made to expose the arch of the atlas, occipital bone, and atlantooccipital membrane. The cisterna magna was percutaneously punctured with a 25-gauge butterfly needle, and 0.30 mL of cerebrospinal fluid was slowly drawn from animals in all groups except G1. The blood drawn from the tail artery was injected into the cisterna magna for approximately 2 min for rats in G3, G4, and G5. The same amount of physiological saline (0.9% NaCl) was injected into the cisterna magna of rats in G2. The rats were then placed in a downward position on an oblique plane for about 15 min to increase blood flow into the basal cisternae. Immediately after SAH, quercetin was administered by gavage to rats in G4 (10 mg/kg) and G5 (50 mg/kg), at intervals of 8 h for 2 days. No drugs were administered to the rats in G3.

All rats were sacrificed at 48th h under appropriate conditions. For histological and morphometric studies, samples from the pons, including the basilar artery (BA), were postfixed in 10% paraformaldehyde for 3 days and then embedded in paraffin.

After removal, all tissues were washed twice with cold saline, placed into glass bottles, labelled and stored at −80 °C until processing. Brain tissues were cut into small pieces with scissors, and then homogenised in 10 volumes of ice-cold 150 mM KCI using a glass-Teflon homogeniser (Ultra Turrax IKA T18 Basic; IKA, Wilmington, NC, USA) for 2 min at 5000 rpm. The homogenate was then centrifuged at 5000 × *g* for 15 min, and the supernatant was used for analysis. High-performance liquid chromatographic (HPLC) analysis was performed (Shimadzu, Kyoto, Japan) with an malondialdehyde (MDA) kit (Immundiagnostik AG, Bensheim, Germany). Measurement of total antioxidant capacity (TAC) (Randox, Crumlin, UK) was performed using a spectrophotometer (8UV-1601; Shimadzu, Kyoto, Japan). 

For histological examination, paraffin-embedded tissue samples from the pons (including the BA) were cut into sections 5 µm thick using a microtome. The sections were stained with haematoxylin and eosin (H&E) and evaluated by light microscopy. Morphometric analyses were performed by the same pathologist using H&E-stained sections obtained from the midportions of the BA. BA wall thickness and diameter were measured using a microscope (DMLB-100S; Leica, Solms, Germany) and Leica QWINPlus v.3.1.0 software. Each BA wall was measured at four points corresponding to 3, 6, 9 and 12 o’clock. Average values for wall thickness and diameter were then calculated. 

Statistical analyses were performed using SPSS (version 19.0; SPSS Inc., Chicago, IL, USA). Descriptive statistics for continuous variables are defined as the median, minimum, and maximum values. The normality of the distribution was examined by the Shapiro–Wilk test. The Kruskal–Wallis test was used for comparison of variables between groups. The Mann–Whitney U with Bonferroni correction was used in subgroup comparisons between two groups. In all analyses, P < 0.05 was taken to indicate statistical significance.

## 3. Results

The MDA level and TAC calculated at 48 h for G1, G2, G3, G4, and G5 are shown in u2921. MDA levels were significantly higher in G2 and G3 than in G1 (P < 0.05). The MDA levels in G4 and G5 were significantly decreased in comparison with G2 (P < 0.05, G4–G2; P < 0.05, G5–G2). No significant differences in MDA level were found between G2 and G3 or among G1, G4, and G5. There were no statistically significant differences in TAC between the groups (P > 0.05).

**Figure 1:**
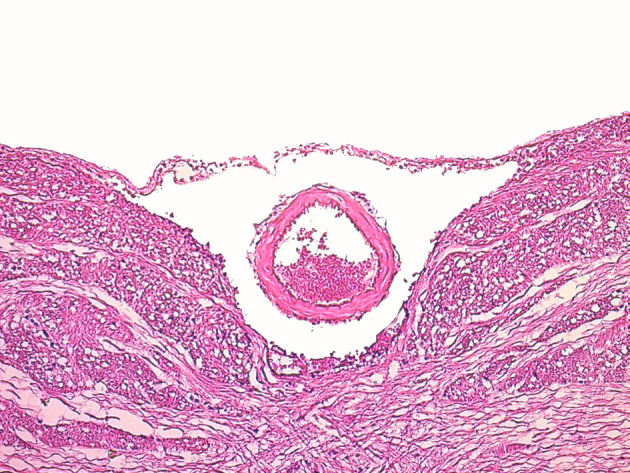
Microscopic view of normal basilar artery in G1 (H&E; X100).

**Figure 2:**
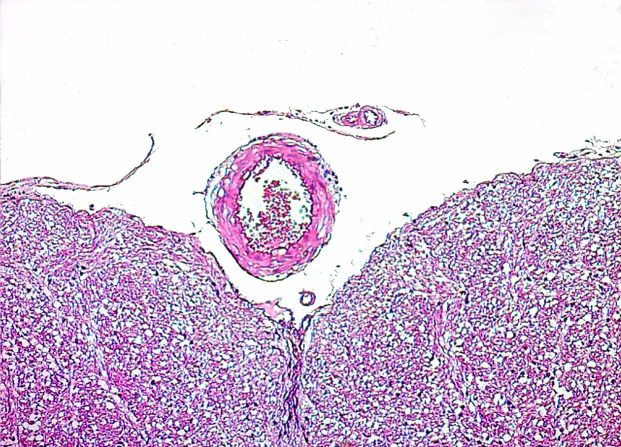
Microscopic view of increase in basilar artery wall thickness and luminal narrowing in G2 compared to G1 (H&E;X100).

**Figure 3:**
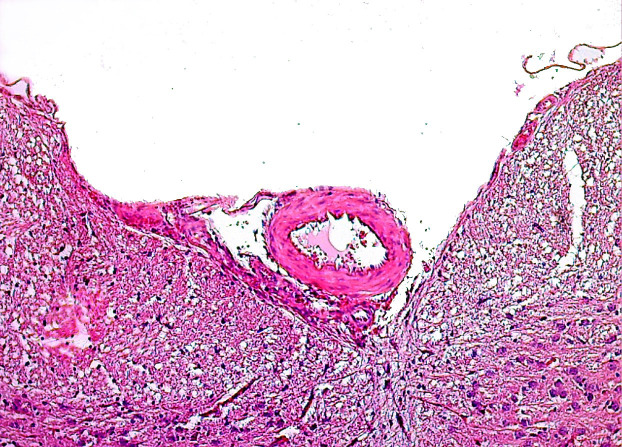
Microscopic view of increase in basilar artery wall thickness and luminal narrowing in G3 compared to G1 (H&E; X100).

**Figure 4:**
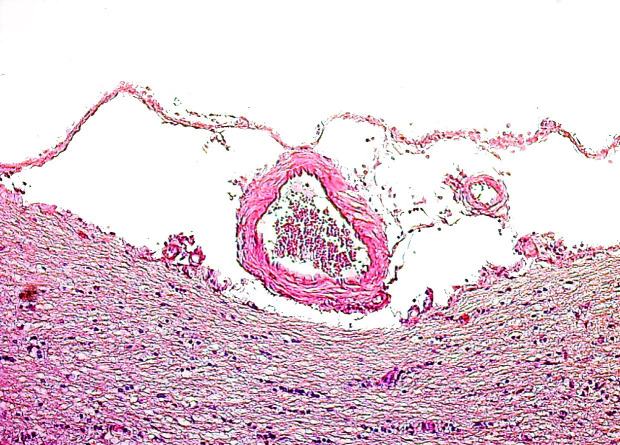
Microscopic view of decrease in basilar artery wall thickness and increase in luminal narrowing in G4 compared to G2 (H&E; X100). No statistically significant difference was observed in basilar artery wall thickness between G4 and G3, but basilar artery luminal diameters were markedly wider in G4.

**Figure 5:**
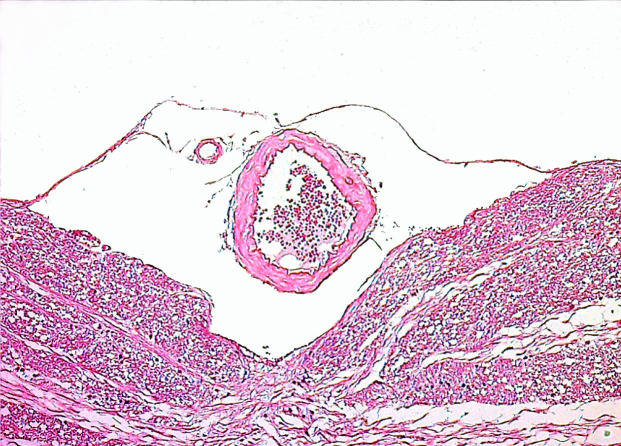
Microscopic view of decrease in basilar artery wall thickness and increase in luminal narrowing in G5 compared to G2 (H&E; X100). No statistically significant difference was observed in basilar artery wall thickness between G5 and G3, but basilar artery luminal diameters were markedly wider in G5.

Histologically, the structure of the BA in G1 was consistent with normal rat cerebral arteries, with a continuous monolayer of endothelium, thin internal elastic lamina and concentrically oriented layers of smooth muscle cells surrounding the intima. In G2 and G3, there was some degree of endothelial swelling with focal desquamation, increased undulation of the internal elastic lamina, markedly thickened arterial walls and luminal narrowing. In G4 and G5, in addition to the endothelial swelling with focal desquamation and distortion of some arteries, the BA walls were thinner and the lumens were more dilated compared to those in G2. The luminal diameters were markedly larger in G4 and G5 than in G3. However, compared with G3, there were no statistically significant differences in arterial wall thickness in G4 and G5 (Figures 1–5).

The results of statistical analyses of the morphometric data for BA wall thickness and luminal diameter are shown in Table 2. Statistically significant differences were observed between G1 and G2, G1 and G3, G2 and G4, and G2 and G5 (P < 0.05).

**Table 1:** Effects of quercetin on MDA and TAC of rat brain tissue following experimental subarachnoid haemorrhage.

Group	n	MDA (nmol/g tissue)	TAC (mmol/g tissue)
G1	8	144.32 ± 6.08	0.25 ± 0.05
G2	8	268.16 ± 27.50	0.23 ± 0.02
G3	8	311.59 ± 11.70	0.17 ± 0.02
G4	7	165.02 ± 10.87	0.20 ± 0.02
G5	7	153.10 ± 12.72	0.23 ± 0.02

Data are mean values ± standard deviation.

**Table 2:** Effects of quercetin on mean luminal diameter and wall thickness of the rat basilar artery following experimental subarachnoid haemorrhage.

Group	n	Mean luminaldiameter (μm)	Mean wallthickness (μm)
G1	8	109.75 ± 1.66	24.06 ± 1.12
G2	8	91.87 ± 2.47	29.56 ± 1.51
G3	8	71.34 ± 1.82	31.90 ± 1.72
G4	7	97.81 ± 4.60	26.96 ± 1.87
G5	7	105.71 ± 3.91	22.53 ± 1.24

Data are mean values ± standard deviation.

There were no significant differences in BA thickness between G3 and G4 or between G3 and G5, but G4 and G5 showed larger luminal diameters than G3 (P < 0.05). There were no significant differences in BA thickness or luminal diameter between G4 and G5.

## 4. Discussion

Subarachnoid haemorrhage is defined as blood between the arachnoid membrane and pia membrane [6]. Aneurysmal haemorrhage has the highest incidence in non-traumatic SAH [6,7]. Blood in the subarachnoid space has an irritant effect, which leads to vasospasm by activating antiinflammatory mechanisms [7]. Early brain injury, seizures, CV, and delayed cerebral ischaemia are important factors contributing to the poor prognosis of SAH [1]. As CV is the leading cause of morbidity and mortality, there have been many experimental animal studies to develop effective treatments [6,7]. However, there is still only limited information about the underlying mechanism of CV. Current treatments for CV following SAH include triple-H therapy (hypertension, hypervolemia, haemodilution), prophylactic hyperdynamic postoperative fluid therapy, and drug therapy [1,6]. As these SAH treatments have not been definitely proven to prevent CV, a number of experimental treatment strategies have been proposed, such as antioxidant drug therapy.

Imbalance in the normal redox state of cells occurs due to oxidative stress and can cause toxic effects through the production of free radicals [8]. Although there are many different mechanisms of free radical production after SAH, autooxidation of haemoglobin is thought to be the main source as soon as it is released into the subarachnoid space [9]. SAH induces the enzymes responsible for reactive oxygen species (ROS) and inhibits the intracellular antioxidant systems [1]. 

Free radicals and peroxides are ROS that trigger oxidative brain injury after cerebral haemorrhage by damaging cellular proteins, lipids and DNA [4]. In addition, glial cells play a role in the development of inflammatory neurodegeneration, and the major intermediate of glial-induced neurotoxicity is the generation of excessive NO, which can induce neuronal cell damage by disrupting the function of the neuronal mitochondrial electron transport chain [8]. 

Quercetin treatment was reported to have potential benefits for conditions involving increased oxidative stress associated with mitochondrial dysfunction [10]. In the planning stages of this study, we expected to observe the antioxidant efficacy of quercetin in an experimental rat SAH model. In a similar study, Dong et al. investigated the dose-dependent effect of quercetin against oxidative stress and brain oedema in an experimental rat model of subarachnoid haemorrhage [4]. 

Cho et al. examined the effects of quercetin against neuronal cell damage and matrix metalloproteinase (MMP)-9 activity after transient global brain ischaemia [11]. Pretreatment of primary hippocampal cultures with quercetin significantly attenuated amyloid b-induced cytotoxicity, protein oxidation, lipid peroxidation, and apoptosis [12]. Quercetin plays an important role in altering the progression of neurodegenerative diseases by its protective effect against oxidative stress [7].

In the present study, the significant decreases in MDA levels observed in G4 and G5 suggested that quercetin may be useful as an alternative antioxidant agent in the treatment of CV following SAH.

The potent ROS scavenging activity of quercetin is attributable to its high number of hydroxyl substitutions, which confer electron-donating ability [13]. In addition to its free radical scavenging effect, quercetin intensifies other antioxidant mechanisms against oxidative stress, such as the chelation of iron and calcium, inhibition of lipid peroxidation and inhibition of nitric oxide synthase [14,15].

Oxyhaemoglobin-induced free radical products and lipid peroxidation are the triggers most strongly implicated in the pathogenesis of CV [2]. 

Stimuli for contraction may include alterations in the balance between vasodilator and vasoconstrictor substances normally produced in the arterial wall, such as prostacyclin, NO, endothelins, etc. [2,16]. 

The increased thickening of arterial walls and narrowing of the luminal diameter are considered signs of vasospasm [1]. In the present study, BA wall thickening increased after SAH and mean luminal diameter values decreased. Significant improvements in histopathological parameters were observed following treatment with low and high doses of hesperidin. In the SAH + low hesperidin and SAH + high hesperidin groups, the mean values of BA wall thickening and luminal diameter were similar to those in the control group. 

Although many studies have demonstrated the protective effects of quercetin against oxidative stress and brain oedema, there have been few reports regarding its effects on CV [4]. Therefore, we investigated the effects of this agent on vasospasm in neural tissue in an experimental SAH model. However, this study had some limitations in that the number of animals was small and the study period was short. Further studies on SAH with quercetin as an antioxidant and vasodilator are required. 

In conclusion, this is the second study to evaluate the possible neuroprotective effects of quercetin on CV in an experimental SAH model. We demonstrated that administration of quercetin induces dose-dependent vasodilatation of BA, which could yield neuroprotective effects. In addition, quercetin appears to be involved in relieving oxidative damage, possibly by reducing lipid peroxidation and/or by increasing the total antioxidant capacity, which may contribute to vascular dilation. Further studies are needed to confirm these results and to elucidate the possible biochemical mechanisms underlying the preventive effect of quercetin on CV.

## Conflicts of interest

The authors certify that they have NO affiliations with or involvement in any organization or entity with any financial interest (such as honoraria; educational grants; participation in speakers’ bureaus; membership, employment, consultancies, stock ownership, or other equity interest; and expert testimony or patent-licensing arrangements), or nonfinancial interest (such as personal or professional relationships, affiliations, knowledge or beliefs) in the subject matter or materials discussed in this manuscript “Neuroprotective effects of quercetin on cerebral vasospasm following experimental subarachnoid haemorrhage in rats”.
